# Microbiota-Associated Metabolites and Related Immunoregulation in Colorectal Cancer

**DOI:** 10.3390/cancers13164054

**Published:** 2021-08-12

**Authors:** Yan Chen, Ying-Xuan Chen

**Affiliations:** Division of Gastroenterology and Hepatology, Key Laboratory of Gastroenterology and Hepatology, Ministry of Health, State Key Laboratory for Oncogenes and Related Genes, Shanghai Institute of Digestive Disease, Renji Hospital, School of Medicine, Shanghai JiaoTong University, Shanghai 200001, China; chenyan_sjtu@sjtu.edu.cn

**Keywords:** microbiota, metabolites, immunity, inflammation, colorectal cancer

## Abstract

**Simple Summary:**

In the past decade, the interaction between intestinal microbiota and colorectal cancer has been an active research area. Microbial metabolites, which could act locally and systematically, have a significant impact on the development of colorectal cancer, especially by inciting immune responses. In this paper, we systematically reviewed the recent insights on microbial metabolites and their immunoregulation on colorectal cancer and discussed the controversial role of some metabolites, hoping to provide a different understanding of the role of bacterial metabolites in colon carcinogenesis.

**Abstract:**

A growing body of research has found close links between the human gut microbiota and colorectal cancer (CRC), associated with the direct actions of specific bacteria and the activities of microbiota-derived metabolites, which are implicated in complex immune responses, thus influencing carcinogenesis. Diet has a significant impact on the structure of the microbiota and also undergoes microbial metabolism. Some metabolites, such as short-chain fatty acids (SCFAs) and indole derivatives, act as protectors against cancer by regulating immune responses, while others may promote cancer. However, the specific influence of these metabolites on the host is conditional. We reviewed the recent insights on the relationships among diet, microbiota-derived metabolites, and CRC, focusing on their intricate immunomodulatory responses, which might influence the progression of colorectal cancer.

## 1. Introduction

The microbiome contains a metagenome 100 times larger than that of the human host [1], playing an increasingly critical role in human health, including digesting indigestible macronutrients and producing vitamins, defending against pathogens, and maintaining immune homeostasis [2]. Increasing data suggest that the microbiota has an impact on the etiology of cancer [3]. The large intestine is where humans are most exposed to microorganisms; therefore, it is not surprising that the interplay between the gut microbiome and colorectal cancer (CRC) has been an active research field.

CRC is one of the most common cancers and the second leading cause of cancer-related deaths worldwide [4]. The established risk factors of CRC include genetic mutations, inflammation, microbiota, and diet [5,6,7,8]. Although a direct causal relationship between the microbiota and CRC remains a matter of debate, it is proposed that bacteria can function as a ‘driver’ in tumorigenesis, complementing the genetic ‘adenoma–carcinoma sequence’ model partly through the production of DNA-damaging compounds and persistent inflammation [9]. CRC is closely associated with a western high-fat, low-fiber diet [6,10,11]. Diet can influence the composition and metabolism of the intestinal flora (Box 1), which helps to produce metabolites that link the microbiota with the host by reprogramming enterocyte metabolism [12], regulating immune responses [13], and impacting tumor formation [3]. Here, we systematically review the latest insights regarding the role of the common metabolites and their elicited downstream responses, especially the immune-related regulation, in CRC development, which deserves further investigation, intending to improve our understanding of CRC and the future development of therapy.

Box 1 **Diet impacts the configuration of gut microbiota.**

The large intestine is characterized by a moderately acidic pH and a slow flow rate; therefore, it has the most considerable microbial flora. Apart from Firmicutes and Bacteroidetes, Actinobacteria, Proteobacteria, and Verrucomicrobia account for the most significant proportion of the microbiota [14]. Sequencing technologies confirmed a higher abundance of SCFAs-producing bacteria, such as *Lachnospira* species, and a high diversity of gut microbiota in people with a traditionally high-fiber diet. In contrast, disease conditions and an industrialized diet are linked to decreased diversity [10,15,16]. Further, long-term dietary intake results in remarkable changes of the microbiome in structure and activity, but also short-term changes in diet induce rapid changes [17,18]. Researchers found that the abundance of microorganisms that could tolerate bile, such as *Bilophila wadsworthia*, increased in the animal-based diet, whereas the abundance of Firmicutes, which could ferment complex carbohydrates, decreased.

## 2. Microbiome-Associated Metabolites, the Immunoregulation, and CRC

Gut microbes can affect the occurrence and development of CRC in multiple different ways. A chronic inflammatory response is a critical factor in the neoplastic process, not only for colitis-associated cancer (CAC) but also for sporadic and familial CRC, explaining why nonsteroidal anti-inflammatory drugs (NSAIDs) can reduce the risk of CRC by 40–50% [7]. Once the intestinal structural barrier is impaired, colonocytes are continuously exposed to microorganisms and their metabolites. Such sustained stimulation of the immune responses might induce chronic mucosal inflammation, which is now widely accepted to promote the neoplastic transformation of the intestinal epithelium [19]. Interestingly, instead of simply escaping the immune clearance to persist in the intestine, the microbiota has complex interactions with colonocytes and resident immune cells through their metabolites [20]. Noteworthily, inflammation, the microbiota, or their metabolites alone are necessary, but insufficient, to trigger the initiation of tumors [21]. However, the intricate interplay among the intestinal flora, metabolites, chronic inflammation or immune responses, host genetics, and essential environmental factors jointly contribute to the development of colon tumors [15]. Metabolites produced by microbiota from a balanced diet serve as essential signals that continuously maintain the epithelial barrier and immune homeostasis, thus exerting protective effects against carcinogenesis. In contrast, harmful metabolites from an unbalanced diet may promote cancer progression.

Although most microbial-derived metabolites that influence the immune responses remain unknown, some related molecules have been identified and studied intensively. To a large extent, the frequency and quantities of these metabolites are diet-dependent and are related to polysaccharide metabolism, protein metabolism, and fat metabolism that the gut microbiota participates. Some representative metabolites associated with CRC and their related mechanisms, especially those implicated in immune regulation, are discussed in detail.

### 2.1. Short-Chain Fatty Acids

Short-chain fatty acids (SCFAs), principally acetate, propionate, and butyrate, are derived from microbial fermentation of dietary fiber [19]. The microbial biotransformation of dietary fiber to monosaccharides in the gut involves numerous intermediate steps (Box 2), mediated by specific gut microbiota members. For instance, most of the acetate and propionate are provided by Bacteroidetes, while butyrate is primarily produced by Firmicutes [22].

Box 2 **Metabolism of SCFAs.**

As the most abundant SCFA, acetate is produced by most enterobacteria from pyruvate via acetyl-CoA [19] and also via the Wood–Ljungdahl pathway by acetogens [23]. As for propionate, it is mostly formed from phosphoenolpyruvate through the succinate pathway by Bacteroidetes and Firmicutes, such as *Phascolarctobacterium succinates* and *Veillonella* spp. [19]. Additionally, using lactate and deoxyhexose sugars as precursors, propionate can be synthesized through the acrylate pathway and the propanediol pathway, respectively [23]. The third major SCFA, butyrate, is mainly generated from Firmicutes via the butyryl-CoA: acetate CoA transferase enzyme [24]. Butyrate is preferred by gut epithelium cells as an energy source, which bridges well established interactions between the microbiota and host health, especially colonic mucosal health [25]. Interestingly, those metabolites can participate in microbial cross-feeding dynamics. Specifically, *Faecalibacterium prausnitzii* can utilize acetate produced by *Bacteroides thetaiotaomicron* as a substrate [20,26]. Some microbes, such as *Eubacterium hallii*, can produce butyrate using both lactate and acetate as substrates, thereby preventing the accumulation of lactate and maintaining the homeostatic balance of the intestines [23].

Acetate, propionate, and butyrate are readily absorbed by the host; however, their subsequent distribution and influences differ. Butyrate is consumed locally as the preferred energy source of colonocytes, whereas the other two absorbed SCFAs are drained into the portal vein. Most propionate metabolism occurs in the liver, resulting in acetate being the most abundant SCFA in the peripheral circulation [27]. Thus, acetate and propionate have more systemic effects on metabolic syndrome [23], while the butyrate has a significant impact on colon health, especially tumor development.

#### 2.1.1. SCFAs as Signaling Molecules and Related Effects on Immunity

**Figure 1:**
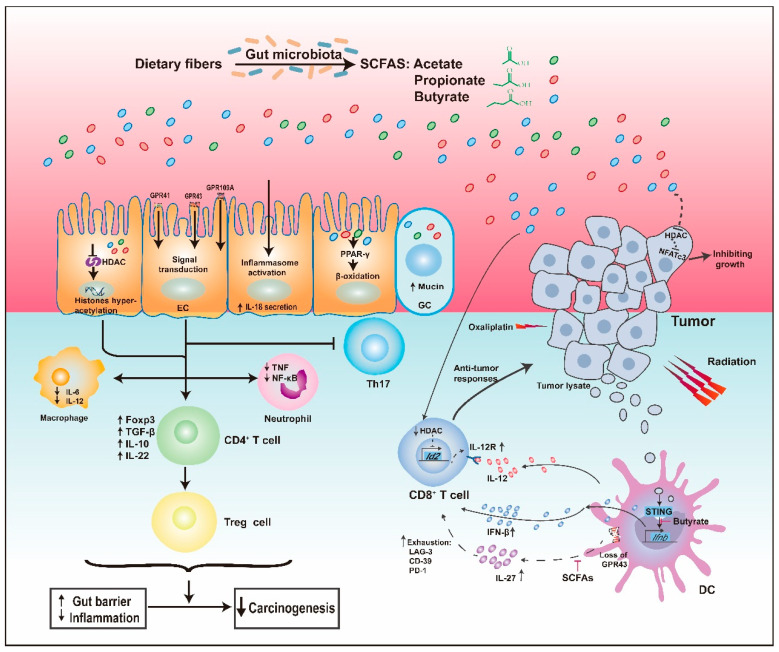
SCFAs and their Immunomodulatory Functions. SCFAs, mainly consisting of acetate, propionate, and butyrate, act as histone deacetylase (HDAC) inhibitors and ligands of G-protein-coupled receptors (GPCRs), leading to the expansion and differentiation of FOXP3+ regulatory T (Treg) cells, accompanied by upregulation of immunosuppressive IL-10 and transforming growth factor-beta (TGF-β), downregulation of proinflammatory cytokines in macrophages and neutrophils, and inhibition of differentiation towards T helper type 17 (Th17) cells, thereby suppressing inflammation and carcinogenesis. Significantly, SCFAs, especially butyrate, could influence the antitumor responses of CD8+ T cells by regulating signaling pathways in dendritic cells (DC), involving IL-12, IL-27, and IFN-β, which also have an impact on the combination therapy of tumor. Additionally, SCFAs directly inhibited tumors by inhibiting HDAC. Regarding their other protective properties, SCFAs could increase the secretion of mucin, enhance epithelial integrity by activating inflammasome, and activate the peroxisome proliferator-activated receptor-γ (PPAR-γ) pathway, which maintains anaerobic conditions. See the text for details. EC, epithelial cell; GC, goblet cell; NF-κB, nuclear factor κB; IFN-β, interferon β.

Increasing evidence shows that SCFAs exert an important influence on preventing carcinogenesis via their anti-inflammatory properties, the induction of apoptosis of cancerous cells, and anti-proliferative activities [28,29,30,31,32]. What are the underlying molecular mechanisms? SCFAs, which act as both extracellular and intracellular signal molecules, play a significant role in the differentiation and functions of host cells, especially immune cells, at least partly through epigenetic modification and receptor-mediated signaling (Figure 1).

##### SCFAs Act as Histone Deacetylase Inhibitors

Once inside the cell, SCFAs can act as inhibitors of histone deacetylases (HDACs) to facilitate the hyperacetylation of histones in colonocytes and immune cells [23]. For example, when exposed to SCFAs, the production of proinflammatory cytokines, such as interleukin-6 (IL-6) and nuclear factor-κB (NF-κB), in mononuclear cells and macrophages decreased, similar to the behavior observed when exposed to HDAC inhibitors [33,34]. Propionate and butyrate also block the differentiation of bone marrow stem cells into dendritic cells (DCs) via HDAC inhibition, thereby reducing undue inflammatory responses [35].

Apart from their effects on innate immune cells, SCFAs also influence the homeostasis of peripheral T cells via HDAC inhibition, particularly facilitating the differentiation of regulatory T cells (Tregs), which is essential to the containment of intestinal inflammation and subsequent tumorigenesis [36,37,38]. Specifically, by promoting hyperacetylation at the promoter and conserved non-coding sequence 1 (CNS1) of the forkhead box P3 (*FOXP3*) locus, butyrate and propionate increased the expression of the *FOXP3* gene, expanded the peripheral pool size of colonic $\mathrm{FOXP}3^{+}$ Tregs, and induced functional IL-10-producing colonic Tregs [37,39]. Notably, acetate, previously not recognized as an inhibitor of HDAC (HDACi), was also found to inhibit HDACs, but only in active immune responses [40]. Significantly, He et al. recently report that butyrate directly enhanced cytotoxic CD8^+^ T cell response through ID2-dependent IL-12 signaling via its HDAC inhibitory activity, thus promoting the antitumor efficacy of oxaliplatin and anti-PD-L1 therapy [41].

SCFAs, as HDAC inhibitors, can also act directly on cancer cells. The metabolic products of *Faecalibaculum rodentium*, mainly SCFAs, directly inhibited colon tumor growth of $Apc^{Min/+}$ mice by downregulation of calcineurin-mediated activation of the NFATc3 in an HDACi-dependent manner [32].

##### SCFAs as GPCR Ligands

Extracellular SCFAs can affect the immune system via meaningful interactions with some G-protein-coupled receptors (GPCRs). There are three main types of GPCRs related to SCFAs, GPR41 (FFAR3), GPR43 (FFAR2), and GPR109A [23], which are expressed on a variety of host cells, including colonocytes and immune cells [37,42]. GPR43 has a higher affinity for acetate and propionate; the ligands of GPR41 are mainly propionate and butyrate [19], while GPR109A only interacts with butyrate and niacin [43]. Smith et al. [36] found that acetate and propionate could induce Tregs in a GPR43-dependent manner to protect against colitis. However, there are some doubts about the GPCR-based mechanism because some studies indicated that T cells did not functionally express GPR41 and GPR43 [40]. Nevertheless, Sivaprakasam et al. [44] confirmed the critical role of SCFAs-GPR43 interaction in the control of inflammation and carcinogenesis. They found that GPR43 was downregulated in human colon cancer tissues compared with that in matched healthy tissues. Consistent with that, GPR43^−/−^ mice suffered more severe colitis and increased numbers of polyps, even on a fiber-plus supplemented diet. In contrast, the expression of inflammatory cytokines, including IL-1β and IL-17A, and tumor loads in the colons of wild-type mice significantly decreased, accompanied by an increase in *Bifidobacterium* species [44]. As for the possible protective mechanisms, for one thing, the expression of GPR43 is necessary for SCFAs-mediated downregulation of the inflammatory response of innate immune cells, such as the infiltration and activation of neutrophil [45]; for another, the activation of GPR43 on the DCs decreased frequency of IL27-producing DCs, which could conduce to the CD8^+^ T-cell exhaustion, and reduced the number of colon tumors in the $Apc^{Min/+}$ mice treated with DSS [46]. Butyrate-mediated activation of GPR109A could upregulate anti-inflammatory effector molecules in colonic DCs and macrophages, which promoted the differentiation of IL-10-producing CD4^+^ T cells and Tregs, and inhibited the development of IL17-producing T cells, thereby preventing colitis and colon carcinogenesis [43]. Besides, the butyrate-GPR109A signaling pathway could block the activation of NF-κB and induce apoptosis in a manner independent of HDAC inhibition [47].

Indeed, sometimes, SCFAs can act as both HDAC inhibitors and GPCR ligands simultaneously. A recent study showed that butyrate could promote IL-22 secretion from CD4^+^ T cells through both GPR41 and inhibition of HDAC to protect intestines from inflammation [48]. Although there is a long way to go from bench to bedside, some findings from animal models have been tested in clinical experiments. Patients with mild-to-moderate ileocolonic Crohn’s disease, the critical risk factor for CRC, were supplemented with 4 g of butyrate daily. After eight weeks, 69% of the patients responded to the treatment, accompanied by downregulation of mucosal inflammatory cytokines [49]. Furthermore, a recent study also found that oral supplementation with butyrate could ease the inflammatory phenotype of monocytes in the blood [50].

#### 2.1.2. Other Antitumor Properties of SCFAs

A healthy intestinal epithelial barrier is undoubtedly crucial because of its functions of maintaining mucosal immunity and preventing the translocation of bacteria and bacterial components. In contrast, a leaky barrier permits an increased level of blood lipopolysaccharide (LPS), which triggers continuously low-grade inflammation [8]. SCFAs can enhance the gut epithelial barrier function, for example, by increasing the secretion of mucus. *Bacteroides thetaiotaomicron*, which produces high levels of acetate, increased mucus production by inducing goblet cell differentiation and the expression of mucus-related genes [26]. Studies also proposed that SCFAs activated the NLR family pyrin domain containing 3 (NLRP3) inflammasome, thereby upregulating the secretion of IL-18, which is protective for epithelial integrity, and ameliorating DSS-induced colitis [51]. Moreover, certain strains of *Bifidobacterium longum*, which are high producers of acetate, defended against fatal infection with enterohaemorrhagic *Escherichia coli* O157:H7 by enhancing epithelial integrity and inhibiting the translocation of lethal toxins into the systemic circulation [52].

Besides, SCFAs have other significant antitumorigenic effects. Intestinal expression of the vitamin D receptor could be restored by butyrate to reduce dysbiosis. The autophagy of butyrate-activated Paneth cells was beneficial to suppress DSS-induced inflammation [53]. Activation of PPAR-γ by butyrate promoted β-oxidation to maintain anaerobic conditions, contributing to intestinal homeostasis [54].

#### 2.1.3. The Butyrate Paradox

Mounting evidence has shown that butyrate is beneficial to protect against CRC; however, some studies reached the opposite conclusion called the “butyrate paradox” [55]. Indeed, butyrate can either enhance or dampen tumor-suppressive responses depending on a specific treatment. For example, in both MC38 and B16F1 tumor models, butyrate abrogated the anti-cancer effect of ionizing radiation by inhibiting the upregulation of STING-activated IFN-I in DCs, which is required for tumor-specific cytotoxic T cell function [56]. As we discussed earlier, SCFAs represented by butyrate could facilitate the differentiation towards functional Tregs, which are potent anti-inflammatory cells, to suppress the chronic inflammation and subsequent tumorigenesis [43]. However, Tregs and other anti-inflammatory cells also mediate tumor immune evasion. Researchers found that bacterial extracts from the microbiota of hepatocellular carcinoma, containing a high level of SCFAs, elicited an immunosuppressive phenotype, featured by expanding Tregs and diminution of CD8^+^ T cells, which may be related to immunotherapy resistance and poor prognosis [57]. Besides, Belcheva et al. reported that butyrate fueled aberrant hyperproliferation of colon epithelial cells in $Apc^{Min/+}Msh2^{-/-}$ mice [58], which is inconsistent with the situation that in tumor cells, butyrate accumulates as an HDACi because of the Warburg effect [59], thereby contributing to the growth inhibition of tumor cells. Researchers argued that this disparity might result from the host’s genetic background and age [60].

Interestingly, butyrate also impacts tumorigenesis by regulating intestinal stem cells (ISC), the cells of origin of intestinal cancer [61,62]. A recent study showed butyrate suppressed the intestinal stem cell proliferation in crypts surrounding the ulcers in a mouse model of colitis by acting as an HDACi to promote the expression of the negative cell-cycle regulator Foxo3 [63]. Although delaying proper wound repair may cause detrimental influence, butyrate prevents stem cells from dividing following exposure to genotoxic luminal contents after mucosal injury, thereby reducing the risk of cancerous transformation of ISC.

Together, these data suggest that butyrate exerts an intricate influence on the crosstalk between the microbiota and the host, especially the immune system. The difference in conclusions of studies might root in the different dose of butyrate, the host genetic background, the tumor stage, and the specific combination therapy, which indicates that the role of butyrate in tumorigenesis will still require further investigation. More importantly, findings from experimental studies need to be tested in large-scale human studies to seek more therapeutic opportunities.

### 2.2. The Impact of Phytochemicals

In addition to SCFAs, there are some metabolites from phytochemicals of fruit, vegetables, and grains, which have antioxidant, anti-inflammatory, and anticarcinogenic properties [64,65,66]. Studies have suggested that all fermentation products together exert better inhibition on colonic cancerous cells than SCFA mixtures alone [67], demonstrating the importance of phytochemicals. Phytochemicals, present in complex glycosylated forms, mainly accumulate in the large intestinal lumen, where they undergo enzymatic transformation by the gut microbiota, such as dehydroxylation or decarboxylation, to be transformed into more bioactive metabolites [64,68]. As widely distributed phytochemicals, dietary polyphenols, especially those in tea, grapes, and coffee, have been investigated intensively. Recently, the immunoregulation of polyphenols related to tumorigenesis is intriguing. It is reported that the tea extracts significantly decreased the production of proinflammatory cytokines (IL-6 and tumor necrosis factor-α (TNF-α))and increased the anti-inflammatory IL-10 in RAW264.7 macrophages and colitis mice [69]. Additionally, ellagitannin-rich cloudberries feeding decreased both the size and number of adenomas in $Apc^{Min/+}$ mice, along with a smaller ratio of intraepithelial to all mucosal CD3^+^ T lymphocytes than that in the control group and attenuation of the mucosal inflammation [70]. In Azoxymethane (AOM)/Dextran sodium sulfate (DSS)-treated mice, the commonly used CAC mouse model, cocoa reduced the colon tumor burden, accompanied by decreased macrophage infiltration and inhibition of IL-6/STAT3 signaling pathways [71].

In addition to suppressing inflammation, polyphenols also prevent tumor-induced dysfunction of effector T cells and restore the immunological surveillance, thus repressing carcinogenesis [72]. However, the antitumor activity of polyphenols seemly depends on settings. Researchers discovered that treatment with gallic acid strikingly facilitated tumorigenesis in the proximal gut of $Apc^{Min/+}$ mice that express mutant p53, not wild-type p53 [73]. Coincidently, AOM/DSS mice treated with *Streptococcus gallolyticus* could degrade tannin to gallic acid and had more colorectal tumors with high malignancy. Mechanistically, *Streptococcus gallolyticus* selectively recruited tumor-infiltrating myeloid cells and thus maintained an immune-suppressive microenvironment [74]. Nevertheless, whether gallic acid mediated the tumor-promoting effect of *Streptococcus gallolyticus* will need further exploration.

There is an interactive relationship between intestinal flora and phytochemical-derived metabolites. Specific bacterial species promote the biotransformation of phytochemicals, which in turn regulate the composition of the gut microbiota and related microbial metabolism [75]. For instance, supplementation with black raspberry anthocyanins reversed the unbalanced composition of the gut microbiota in AOM/DSS-treated mice, inhibiting the pathogenic *Desulfovibrio* sp. and *Enterococcus* spp., and restoring probiotics such as *Faecalibacterium prausnitzii* and *Lactobacillus*, thus partly preventing the carcinogenesis [76]. Oxyberberine, transformed from berberine by intestinal microflora, appreciably suppressed TLR4-MyD88-NF-κB innate immune signaling pathway and ameliorated DSS-induced colitis, implicated in normalizing the dysbacteriosis [66]. Interestingly, high-molecular-weight polysaccharides (>300 kDa) derived from the fungi *Hirsutella sinensis* selectively enriched *Parabacteroides goldsteinii* in high-fat diet (HFD)-fed mice. Of note, oral administration of HFD-fed mice with live *P. goldsteinii* reproduced the benefits of *H. sinensis*, including augmenting intestinal integrity and reducing levels of inflammation [77]. While shaping the landscape of gut microbiota, phytochemical-derived metabolites also influence its metabolism, for example, by promoting the production of SCFAs. More specifically, indole-3-carbinol selectively increased butyrate-producing *Roseburia* through up-regulating IL-22. The increment of butyrate effectively suppressed the proinflammatory Th17 cells and induced anti-inflammatory Tregs in the mesenteric lymph nodes of colitis mice [78]. Besides, phytochemical-derived compounds could impact the development of cancer by altering the colonic bacterial enzyme activities. Rosmarinic acid, one of the major components of polyphenol, significantly decreased the activities of bacterial β-glucosidase and mucinase, which respectively hydrolyzed 1,2-dimethylhydrazine (DMH) to toxic methylazoxymethanol and degraded protective mucin, thereby restraining the colon carcinogenesis in DMH treated rats [79]. Collectively, phytochemicals perform protection against colonic inflammation and tumorigenesis together with SCFAs, consolidating the evidence that a balanced diet containing fruits and vegetables is beneficial to health.

### 2.3. Proteolytic Fermentation and Related Metabolites

**Figure 2:**
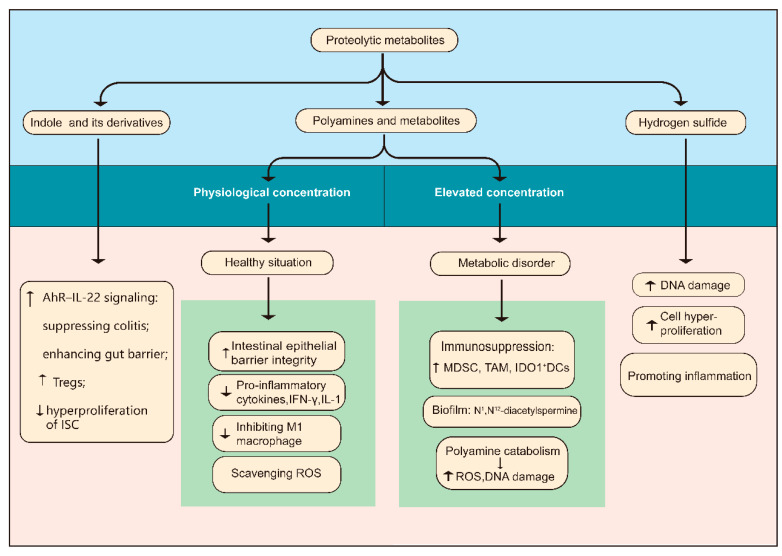
Immunomodulatory metabolites from dietary protein. There are some representative protein-derived metabolites related to immunoregulatory functions, comprising indole and its derivates, polyamines, and hydrogen sulfide. AhR, aryl hydrocarbon receptor; ISC, intestinal stem cell; ROS, reactive oxygen species; RNS, reactive nitrogen species; MDSC, myeloid-derived suppressor cells; TAM, tumor-associated macrophages; IDO1, indoleamine 2,3-dioxygenase 1.

Generally, elevated proteolytic fermentation of a high-protein diet in the gut produces some potentially toxic by-products, such as amines and hydrogen sulfide [15,80]. In the following part, we focus on some representative metabolites and their controversial immunoregulation on the progression of colonic carcinogenesis (Figure 2).

#### 2.3.1. Indole and Its Derivatives

Indole and its derivatives are major bacterial metabolites produced from diet tryptophan. Indole can be transformed by a variety of Bacteroides and Enterobacteriaceae with tryptophanase activity, while indole derivatives are only produced by a few commensal species, including *Peptostreptococcus* spp. and *Lactobacillus* spp. [81]. Both of them are the main ligands for the aryl hydrocarbon receptor (AhR) [42]. Being a cytosolic transcription factor, AhR is induced by ligands and expressed on epithelial cells, immune cells, and tumor cells [13]. Given that AhR signaling is a pivotal component of the immune response at barrier sites, the interplay between the metabolites and AhR has a significant influence on maintaining intestinal homeostasis, inhibiting infection with pathogens [82], and ameliorating DSS-induced colitis [83]. Those protective mechanisms are mainly mediated by IL-22, for example by reprogramming the differentiation of CD4^+^ T cells to Tregs [84] and enhancing the gut barrier function [85,86]. Researchers found that mice lacking *Card9*, a gene related to susceptibility to inflammatory bowel disease (IBD) in humans and encoding caspase recruitment domain family member 9, were more prone to develop colitis accompanied by dysbiosis, deficient indole derivatives, and decreased IL-22 levels, which was similar to IBD patients deficient in CARD9 [87].

AhR activation also exerts a cancer-preventive effect that does not rely solely on suppressing inflammation. Indole-3-aldehyde produced by *Lactobacillus reuteri* D8 activated STAT3 to restore the function of Lgr5^+^ ISC via AhR-IL-22 signaling, thus recovering the regeneration of intestinal epithelial following inflammatory assault [88]. Furthermore, IL-22 also protected stem cells against malignant transformation in the presence of genotoxic stress via regulating components of the DNA damage response [89]. Additionally, mice exhibiting dysregulated AhR signaling developed large tumors throughout the colon within 4 months of AOM application, whereas no tumors were observed in wild-type mice. However, the effective activation of AhR by dietary ligands prevented colonic tumorigenesis via restoring the dysregulated Wnt-β-Catenin pathway, which indicated the unrestricted proliferation of intestinal stem cells [82]. Moreover, the interaction between Indole 3-propionic acid (IPA) and the pregnane X receptor likewise contributed to enhancing the gut barrier function and easing intestinal inflammation [90]. Collectively, indole metabolites have protective effects on the development of tumors in the colon in an AhR-dependent manner, including inhibiting inflammation, promoting barrier function, and restraining the hyperproliferation of intestinal stem cells.

#### 2.3.2. Polyamines and Associated Metabolism

Polyamines are arginine derivatives, metabolized from host tissues and the gut microbiota, including putrescine and spermine [19]. Generally, they are implicated in a variety of biological functions. Polyamines maintain intestinal health by enhancing the intestinal barrier through the expression of Toll-like receptor 2, secretion of mucin, and induction of E-cadherin [91,92]. Unexpectedly, Levy et al. found that spermine was overrepresented in the colons of NLRP6-deficient mice, which caused decreased secretion of IL-18, thereby weakening the gut barrier [93]. Additionally, polyamines perform anti-inflammatory functions. Myeloid-specific deletion of ornithine decarboxylase (ODC), the rate-limiting enzyme in polyamine synthesis, promoted M1 immune response and therefore significantly enhanced gastritis during *Helicobacter pylori* infection, but alleviated DSS-induced colitis [94,95]. Consistent with that, polyamines downregulated LPS-induced IL-1 and IFN-γ, upregulated the production of IL-10 in macrophages [96], and suppressed the activation of proinflammatory M1 macrophages [94]. Moreover, as natural reactive oxygen species (ROS) scavengers, they could also protect DNA from oxidative stress [97].

Nevertheless, dysregulated levels of polyamines are related to cell dysfunction. Aberrant polyamine metabolism is linked to cancer development [97]. Elevated polyamine levels in cancer contribute significantly to immunosuppression in the tumor microenvironment (TME), such that the polyamine blocking therapy restricted tumor growth and enhanced the antitumor efficacy of PD-1blockade through increasing tumor-specific cytotoxic T-cells while decreasing myeloid-derived suppressor cells (MDSC) and M2-like tumor-associated macrophages (TAM), which are characterized by high levels of arginase 1 (ARG1) and therefore deprived of arginine that is essential for T cell activation [98,99]. Consistently, loss of ODC in macrophages protected mice from colon carcinogenesis in the AOM/DSS model by increased M1 response against tumors [95]. Besides, polyamines favor the immunosuppressive tumor microenvironment by rendering DCs immunosuppressive dependent on indoleamine 2,3-dioxygenase 1 (IDO1) [100] and promoting M2 polarization in macrophages through inducing mitochondrial oxidative phosphorylation by eIF5A hypusination [101]. Noteworthily, spermidine improved tumor chemotherapy in an immune-dependent fashion by stimulating autophagy [102], which suggests the role of polyamines in tumors varies with conditions.

As for the other effects of polyamines on tumors, Johnson et al. found that upregulation of *N*^1^,*N*^12^-diacetylspermine, the end-product of spermine metabolism, within biofilm-positive colon cancer tissues was further enhanced, indicating that polyamine metabolites are associated with the development of CRC [103]. Although it has been argued that polyamines promote the formation of carcinogenic biofilms [103], the relationship between polyamine metabolites within the biofilm and CRC remains to be further investigated. Another representative example of aberrant polyamine metabolism acting on tumorigenesis is associated with *Enterotoxigenic Bacteroides fragilis* (ETBF), a widely supported contributor to intestinal tumorigenesis [55]. ETBF was reported to be upregulated polyamine catabolism, thereby exaggerating inflammation and tumorigenesis via ROS-related DNA damage [104].

The relationship between polyamines and cancer has been investigated for several decades. Polyamines appear to be both anti-carcinogenic and pro-tumorigenic, depending on the context, such as the concentration, the products of metabolism, the developmental stage of the tumor, and combination therapy. Based on the important role of the polyamine pathway in carcinogenesis, targeting it might lead to tumor prevention and curative treatment. Certainly, the intricate interplay between microbial and host polyamine metabolism and the development of colon cancer deserves further exploration.

In addition to metabolites discussed above, Hydrogen sulfide (H_2_S), produced by thiogenic bacteria by reducing inorganic sulfur or fermenting sulfur-containing amino acids, also contributed to colon carcinogenesis partly through its proinflammatory property [105]. For instance, one study discovered a significant expansion of *Bilophila wadsworthia* and more severe inflammation in IL10^−/−^ mice supplemented with taurocholate instead of glycocholate [106], which indicated the important role of taurine-derived sulfide in colitis. 

Taken together, proteolytic metabolites have an immunoregulation effect on the development of CRC. Notably, the effects of a high-protein diet on CRC in humans could be even much more complicated when it comes to the specific sources of protein and chemical reactions of other dietary components consumed in addition to protein. For example, the heme in red and processed meats promoted the formation of carcinogenic N-nitroso compounds and aldehydes and elicited epithelium damage and hyperproliferation, which could be facilitated by H_2_S via breaking the gut mucin barrier [107], thus H_2_S and heme have a synergistic effect on carcinogenesis.

### 2.4. Bile Acid Metabolism

**Figure 3:**
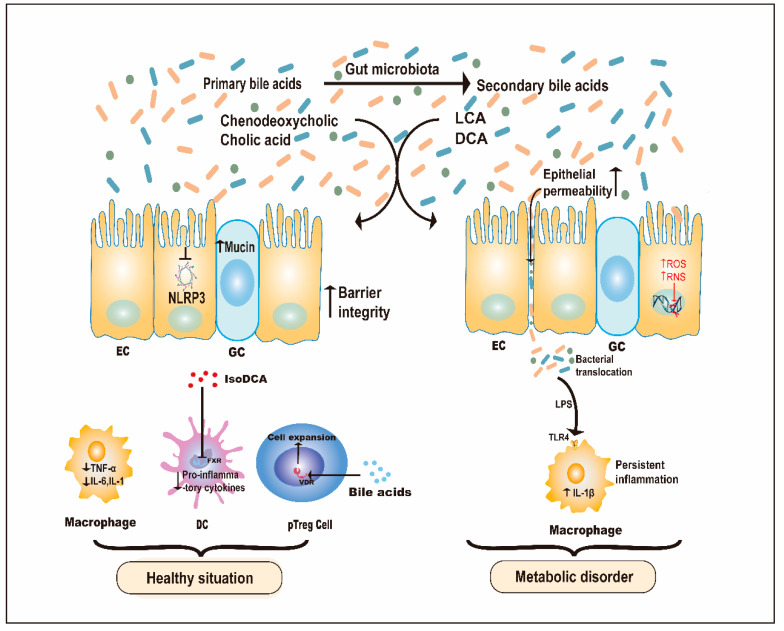
Immunoregulatory effects of bile acids on host cells. Bile acids consist of primary bile acids (such as cholic acid and chenodeoxycholic acid) and secondary bile acids, including deoxycholic acid (DCA) and lithocholic acid (LCA). The latter is metabolized from the former by gut microbiota. In the healthy situation, both types of bile acids can inhibit the activation of the NLRP3 inflammasome in epithelial cells (ECs), downregulate proinflammatory cytokines, such as IL-6 and IL-1, in macrophages and dendritic cells (DCs), and promote peripheral-induced regulatory T (pTreg) cell expansion in a vitamin D receptor (VDR)-dependent manner. IsoDCA, a secondary bile acid, contributes to pTreg cell expansion by counteracting farnesoid X receptor (FXR) in DCs. Additionally, they promote the secretion of mucin in goblet cells (GCs), which helps to enhance the gut barrier. However, when there is a metabolic disorder caused by a high-fat diet, the concentration of bile acids, especially secondary bile acids, are elevated, resulting in a high level of reactive oxygen species (ROS) and reactive nitrogen species (RNS), and increased epithelial permeability, which causes translocation of bacteria and their metabolites and persistent inflammation.

Bile acids (BAs) contain primary bile acids and secondary bile acids (Figure 3), associated with the consumption of a high-fat and low complex carbohydrate diet, and primarily help absorb dietary fats and fat-soluble vitamins [108]. Primary bile acids, mainly including cholic acid (CA) and chenodeoxycholic acid (CDCA), are synthesized from cholesterol within hepatocytes and are secreted after being combined with glycine or taurine. Approximately 5% of them escape enterohepatic circulation and then enter the colon, where they are subjected to microbial metabolism to form secondary bile acids, such as deoxycholic acid (DCA) and lithocholic acid (LCA) [20]. The ratio of primary/secondary BAs depends on the situation. For healthy people, the ratio in the duodenal fluid is about 5 to 6 [109,110], the ratio in feces is around 4, with primary BAs accounting for 80% [111] and the ratio of DCA metabolites to CA metabolites in serum is close to 5 [112]. However, for patients with colorectal cancer or polyps, the proportion of secondary BAs significantly increased [113], especially the LCA. Altogether, the above data might suggest a relationship between CRC and secondary BAs. BAs generally act on receptors, including G protein-coupled bile acid receptor 1 (GPBAR1; also known as TGR5) and the farnesoid X receptor (FXR) [114]. These receptors are highly represented in innate immune cells and have pivotal roles in mediating anti-inflammatory effects, which involve inhibition of the NLRP3 inflammasome; downregulation of proinflammatory cytokines in innate immune cells, partly through inhibiting the NF-KB signaling pathway [114,115,116,117]; and the preservation of the intestinal barrier [118,119]. A recent study confirmed that supplementation with LCA and DCA, which are reduced in pouches of ulcerative colitis (UC), mitigated inflammation, partly relying on TGR5 activation on immune cells [120]. Although the action of BAs on the adaptive immune system remains poorly characterized, researchers found that 3β-hydroxydeoxycholic acid (isoDCA) expanded the population of colonic RORγ+ Tregs by counteracting FXR signaling in DCs, thereby minimizing the severity of colitis. The bile acid–vitamin D receptor signaling axis plays an important role in maintaining immunological balance in the colon [121,122].

Despite the above evidence suggesting that bile acids have anti-inflammatory effects, some studies argue that metabolic disorders of bile acids can contribute to inflammatory diseases and carcinogenesis [123,124,125]. Indeed, African Americans with a higher risk of CRC have a higher concentration of secondary bile acids in their feces in comparison with rural Africans [11,126]. Emerging experimental evidence supports that a high level of secondary bile acids is carcinogenic to the colon, for example, by driving malignant transformations in cancer stem cells [127,128]. As an example, the HFD diet appreciably increased the levels of tauro-β-muricholic acid (T-βMCA) and DCA in $Apc^{Min/+}$ mice, which markedly impaired intestinal integrity and promoted tumor growth. Mechanistically, T-βMCA and DCA antagonized the function of FXR, thus inducing the proliferation and genomic instability in Lgr5-expressing cancer stem cells, fueling the progression of colorectal cancer [129]. BAs-TGR5-SRC/YAP signaling in Lgr5^+^ cells are essential to maintain the regeneration of intestinal epithelium after DSS damage and thus curtailed colitis in mice [130]. Regarding direct cancer-promoting effects of BAs, researchers found that among 18 wild-type mice given DCA orally for 8–10 months, 17 developed colon tumors, of which 10 suffered from cancer, whereas tumor formation could be reversed by the addition of the antioxidant, chlorogenic acid [131]. This was consistent with the fact that the carcinogenic properties of secondary bile acids are implicated in inducing ROS and RNS, thus causing DNA damage and inflammatory damage [132]. Moreover, elevated levels of secondary bile acids or their abnormal proportion are believed to increase epithelial permeability. For instance, upregulation of certain highly hydrophobic secondary bile acid levels and reduction of ursodeoxycholic acid (UDCA) levels is related to epithelial apoptosis [133,134], resulting in bacterial translocation and persistent low-grade inflammation, which eventually promoted carcinogenesis. Besides, DCA at high concentrations (100 µM and greater) was reported to activate the NLRP3 inflammasome and induce high levels of IL-1β in LPS-primed macrophages [135]. 

Taken together, whether bile acids function as carcinogenic agents or tumor-suppressors depends on many factors, such as the concentrations and the specific types of bile acid, the cell type being exposed, and the interactions with other metabolites. Although the complicated roles of bile acids in regulating intestinal immunity and carcinogenesis requires further elucidation, animal experiments have demonstrated that activation of TGR5 and FXR with synthetic agonists, or their reactivation in colon tumors, protected against inflammation and tumorigenesis, which provide good prospects for IBD and CRC therapy [118,136]. 

## 3. Specific Pathogens Associated with CRC

So far, we have discussed the role of bacterial metabolites in the etiology of CRC. Indeed, certain specific microorganisms are involved in promoting CRC. Accumulating metagenomic analyses have shown a higher abundance of *Fusobacterium* (*F.*) *nucleatum* in CRC tissues versus matched normal tissues [137,138]. Moreover, liver metastases of CRC were reported to contain fusobacterium strains and their associated microbiome that are highly similar to the primary tumors [139], suggesting a strong association between *F. nucleatum* and the metastasis of CRC. A previous study argued that *F. nucleatum* could promote inflammation and oncogenesis by activating the β-catenin signaling pathway [140]. Noteworthily, *F. nucleatum* appears to be implicated in reforming the TME. The interaction between Fap2, a microbial protein of *F. nucleatum*, and the inhibitory receptor T cell immunoreceptor with Ig and ITIM domains (TIGIT) of immune cells, and the infiltration of myeloid cells in intestinal tumors of ${\mathrm{Apc}}^{\mathrm{Min}/+}$ mice driven by *F. nucleatum*, could suppress effector killer cells and promote immune evasion, favoring the survival and growth of the tumor [141,142]. Besides, the enrichment of *F. nucleatum* correlated negatively with CD3^+^ T cell density in CRC tissue [143,144]. Other mechanisms contributing to carcinogenesis by *F. nucleatum* are related to promoting proliferation and mediating chemoresistance via activating autophagy [145,146]. 

Although inflammatory response genes such as IL-6, COX-2, and TNF were upregulated in CRC patients with a high abundance of *F. nucleatum* [141], whether *F. nucleatum* contributes to colitis-associated carcinogenesis is controversial because *F. nucleatum* could not exacerbate colitis or inflammation-associated intestinal carcinogenesis [141]. Nevertheless, ETBF and *Escherichia* (*E.*) *coli* are thought to promote colitis-associated cancer. Recently, a metagenomic analysis from four cohorts of patients with CRC found that *Bacteroides fragilis* exists consistently in the gut microbiota across populations [147]. The gene encoding *B. fragilis* toxin (BFT), accounting for ETBF pathogenicity, is more prevalent in the colonic mucosa of patients with CRC [148]. ETBF could rapidly induce colonic inflammation and tumors in a mouse model of multiple intestinal neoplasias depending on IL-17 signaling through inciting the infiltration of immunosuppressive myeloid cells in TME [149,150,151]. *E. coli* adhering to and invading IECs has been observed increasingly in patients with IBD and CRC [152]. The polyketide synthase (PKS) genotoxic island encodes genes that synthesize the colibactin genotoxin, which could cause DNA damage in vivo and in vitro, thereby partly explaining the pro-tumorigenesis role of $pks^{+}\;$*E. coli* [153]. Furthermore, exposure of human intestinal organoids to $pks^{+}\;$*E. coli* induced specific mutational signatures that were also detected in genomes from patients with CRC [154].

Notably, interactions between microorganisms might have a synergistic contribution to inflammation and tumorigenesis. For example, the invading biofilm in the colonic mucosa of patients with familial adenomatous polyposis (FAP) predominantly comprises $pks^{+}$ *E. coli* and ETBF. Colon tumorigenesis was reinforced in tumor-prone mice co-colonized with both strains compared with mono-colonization with either bacterium. Mechanistically, mucus degradation induced by ETBF promoted the colonization of $pks^{+}$ *E. coli*, resulting in enhanced DNA damage, which might contribute to tumorigenesis [153]. 

*Peptostreptococcus* (*P.*) *anaerobius*, significantly enriched in the feces and tissues of CRC patients, has also been investigated. Researchers found that, by acting on TLR2 and TLR4, *P. anaerobius* could facilitate colonic tumorigenesis through promoting cholesterol biosynthesis in a ROS-dependent manner [155]. Beyond that, the surface protein of *P. anaerobius*, putative cell wall binding repeat 2 (PCWBR2) had been identified. Firstly, PCWBR2 directly interacts with α2/β integrin, a receptor generally overexpressed in human CRC tumors and cell lines, which might explain why *P. anaerobius* preferentially colonizes the CRC tumors [156]; and second, *P. anaerobius* activated the α2/β1-PI3K–Akt–NF-κB signaling cascade to trigger the inflammation in $Apc^{Min/+}$ mice. Besides, *P. anaerobius* expanded immune-suppressive MDSCs, TAMs, and granulocytic TANs, to promote tumor progression [156]. Collectively, *P. anaerobius* facilitated the tumor progression by promoting inflammation, modifying the immune cells in TME, and regulating the metabolism of cancerous cells.

## 4. Conclusions and Perspectives

The experimental and clinical evidence discussed above indicates that specific pathogens and microbial metabolites play a critical role in the interactions between the microbiome and immune responses regarding tumorigenesis. Significantly, despite few studies about the role of the microbiota on the efficacy of CRC immunotherapy, researchers found that inosine derived from *Bifidobacterium pseudolongum* activated antitumor T cells via the adenosine A2A receptor, thereby enhancing checkpoint inhibitor immunotherapy [157]. Shalapour and Karin proposed that intestinal barrier disruption was the origin of tumor-promoting inflammation, combined with cancer-initiating mutations, contribute to the formation of a tumor [158]. Correspondingly, the Western diet, with high-fat levels and low complex carbohydrate levels, disturbs gut microbiota homeostasis, resulting in impaired biogenesis of protective metabolites, a compromised gut barrier, continuous inflammation, and eventual carcinogenesis. Except for the microorganisms and metabolites mentioned above, other less investigated bacteria, fungi, archaea, viruses, and metabolites synthesized de novo by gut microbes also influence the host (reviewed elsewhere [10,13]). To give an example, Nod1 stimulation by bacterial peptidoglycan-derived muramyl peptides (MPs) induced monocytic MDSCs infiltration and TAM, which drove carcinogenesis in AOM/DSS treated mice and $Apc^{Min/+}$ mice [159]. The latest studies uncovered that *Streptococcus thermophilus* and *Faecalibaculum rodentium* exerted anti-cancer effects by their metabolites, which reminds us that the role of the bacteria and their metabolites strongly reduced during carcinogenesis is worth exploring to seek therapy opportunity [32,160]. Although there are some controversial findings because of discordance in experimental models, specimens, or contexts, we still see the prospective benefits of screening, diet interventions, therapy, and predicting the prognosis of CRC based on large-scale epidemiological investigations and clinical studies (Table 1).

Although most studies have been well performed, further studies to determine the biological mechanism are needed to go beyond simple correlations. Given that data on diet intervention are primarily derived from observational or retrospective studies, to finally determine causality, further larger-scale longitudinal intervention studies on specific metabolites, as well as new technological advances, are required [13].

## Figures and Tables

**Table 1:** Key Clinical Diet Intervention Associated with Reduced Risk and Improved Survival of CRC.

Studies	Study Design	Sample Size	Main Finding
Bingham et al., 2003 [161]	Prospective cohort study	1065 cancers	Dietary fiber intake was inversely associated with the incidence of CRC; Greatest protection on the left side of the colon
Murphy et al., 2012 [162]	Prospective cohort study	4517 cancers	Total dietary fiber was inversely related to colorectal cancer
Song et al., 2019 [163]	Prospective cohort study	1575 patients with stage I to III CRC	High intake of the total fiber after diagnosis was associated with lower mortality; Cereal fiber was associated with lower CRC-specific mortality
Schmit et al., 2016 [164]	Case-control study	5145 cases and 4097 controls	Coffee consumption was associated with a lower risk of CRC
Hu et al., 2018 [165]	Prospective cohort study	1599 patients with stage I to II CRC	Intake of caffeinated and decaffeinated coffee after diagnosis of CRC was associated with a lower risk of CRC-specific death and overall death
Mackintosh et al., 2020 [166]	Prospective cohort study	1171 advanced or metastatic cancers	In patients with advanced or metastatic CRC, coffee consumption may be associated with a decreased risk of disease progression and death

## References

[1] Thomas S., Izard J., Walsh E., Batich K., Chongsathidkiet P., Clarke G., Sela D.A., Muller A.J., Mullin J.M., Albert K. (2017). The Host Microbiome Regulates and Maintains Human Health: A Primer and Perspective for Non-Microbiologists. Cancer Res..

[2] Lynch S.V., Pedersen O. (2016). The Human Intestinal Microbiome in Health and Disease. N. Engl. J. Med..

[3] Schwabe R.F., Jobin C. (2013). The microbiome and cancer. Nat. Rev. Cancer.

[4] Siegel R.L., Miller K.D., Jemal A. (2020). Cancer statistics, 2020. CA Cancer J. Clin..

[5] Fearon E.R. (2011). Molecular genetics of colorectal cancer. Annu. Rev. Pathol..

[6] Thorburn A.N., Macia L., Mackay C.R. (2014). Diet, metabolites, and “western-lifestyle” inflammatory diseases. Immunity.

[7] Lasry A., Zinger A., Ben-Neriah Y. (2016). Inflammatory networks underlying colorectal cancer. Nat. Immunol..

[8] Cani P.D. (2018). Human gut microbiome: Hopes, threats and promises. Gut.

[9] Tjalsma H., Boleij A., Marchesi J.R., Dutilh B.E. (2012). A bacterial driver-passenger model for colorectal cancer: Beyond the usual suspects. Nat. Rev. Microbiol..

[10] Song M.Y., Chan A.T., Sun J. (2020). Influence of the Gut Microbiome, Diet, and Environment on Risk of Colorectal Cancer. Gastroenterology.

[11] O’Keefe S.J., Li J.V., Lahti L., Ou J., Carbonero F., Mohammed K., Posma J.M., Kinross J., Wahl E., Ruder E. (2015). Fat, fibre and cancer risk in African Americans and rural Africans. Nat. Commun..

[12] Araujo J.R., Tazi A., Burlen-Defranoux O., Vichier-Guerre S., Nigro G., Licandro H., Demignot S., Sansonetti P.J. (2020). Fermentation Products of Commensal Bacteria Alter Enterocyte Lipid Metabolism. Cell Host Microbe.

[13] Rooks M.G., Garrett W.S. (2016). Gut microbiota, metabolites and host immunity. Nat. Rev. Immunol..

[14] Eckburg P.B., Bik E.M., Bernstein C.N., Purdom E., Dethlefsen L., Sargent M., Gill S.R., Nelson K.E., Relman D.A. (2005). Diversity of the human intestinal microbial flora. Science.

[15] O’Keefe S.J. (2016). Diet, microorganisms and their metabolites, and colon cancer. Nat. Rev. Gastroenterol. Hepatol..

[16] Sonnenburg E.D., Sonnenburg J.L. (2014). Starving our microbial self: The deleterious consequences of a diet deficient in microbiota-accessible carbohydrates. Cell Metab..

[17] Smits S.A., Leach J., Sonnenburg E.D., Gonzalez C.G., Lichtman J.S., Reid G., Knight R., Manjurano A., Changalucha J., Elias J.E. (2017). Seasonal cycling in the gut microbiome of the Hadza hunter-gatherers of Tanzania. Science.

[18] David L.A., Maurice C.F., Carmody R.N., Gootenberg D.B., Button J.E., Wolfe B.E., Ling A.V., Devlin A.S., Varma Y., Fischbach M.A. (2014). Diet rapidly and reproducibly alters the human gut microbiome. Nature.

[19] Louis P., Hold G.L., Flint H.J. (2014). The gut microbiota, bacterial metabolites and colorectal cancer. Nat. Rev. Microbiol..

[20] Postler T.S., Ghosh S. (2017). Understanding the Holobiont: How Microbial Metabolites Affect Human Health and Shape the Immune System. Cell Metab..

[21] Slowicka K., Petta I., Blancke G., Hoste E., Dumas E., Sze M., Vikkula H., Radaelli E., Haigh J.J., Jonckheere S. (2020). Zeb2 drives invasive and microbiota-dependent colon carcinoma. Nat. Cancer.

[22] Levy M., Thaiss C.A., Elinav E. (2016). Metabolites: Messengers between the microbiota and the immune system. Genes Dev..

[23] Koh A., De Vadder F., Kovatcheva-Datchary P., Backhed F. (2016). From Dietary Fiber to Host Physiology: Short-Chain Fatty Acids as Key Bacterial Metabolites. Cell.

[24] Louis P., Duncan S.H., McCrae S.I., Millar J., Jackson M.S., Flint H.J. (2004). Restricted distribution of the butyrate kinase pathway among butyrate-producing bacteria from the human colon. J. Bacteriol..

[25] Roediger W.E. (1982). Utilization of nutrients by isolated epithelial cells of the rat colon. Gastroenterology.

[26] Wrzosek L., Miquel S., Noordine M.L., Bouet S., Joncquel Chevalier-Curt M., Robert V., Philippe C., Bridonneau C., Cherbuy C., Robbe-Masselot C. (2013). *Bacteroides thetaiotaomicron* and *Faecalibacterium prausnitzii* influence the production of mucus glycans and the development of goblet cells in the colonic epithelium of a gnotobiotic model rodent. BMC Biol..

[27] Cummings J.H., Pomare E.W., Branch W.J., Naylor C.P., Macfarlane G.T. (1987). Short chain fatty acids in human large intestine, portal, hepatic and venous blood. Gut.

[28] Buda A., Qualtrough D., Jepson M.A., Martines D., Paraskeva C., Pignatelli M. (2003). Butyrate downregulates α2β1 integrin: A possible role in the induction of apoptosis in colorectal cancer cell lines. Gut.

[29] Donohoe D.R., Holley D., Collins L.B., Montgomery S.A., Whitmore A.C., Hillhouse A., Curry K.P., Renner S.W., Greenwalt A., Ryan E.P. (2014). A gnotobiotic mouse model demonstrates that dietary fiber protects against colorectal tumorigenesis in a microbiota- and butyrate-dependent manner. Cancer Discov..

[30] Malcomson F.C., Willis N.D., Mathers J.C. (2015). Is resistant starch protective against colorectal cancer via modulation of the WNT signalling pathway?. Proc. Nutr. Soc..

[31] Tian Y., Xu Q., Sun L., Ye Y., Ji G. (2018). Short-chain fatty acids administration is protective in colitis-associated colorectal cancer development. J. Nutr. Biochem..

[32] Zagato E., Pozzi C., Bertocchi A., Schioppa T., Saccheri F., Guglietta S., Fosso B., Melocchi L., Nizzoli G., Troisi J. (2020). Endogenous murine microbiota member *Faecalibaculum rodentium* and its human homologue protect from intestinal tumour growth. Nat. Microbiol..

[33] Usami M., Kishimoto K., Ohata A., Miyoshi M., Aoyama M., Fueda Y., Kotani J. (2008). Butyrate and trichostatin A attenuate nuclear factor kappaB activation and tumor necrosis factor alpha secretion and increase prostaglandin E2 secretion in human peripheral blood mononuclear cells. Nutr. Res..

[34] Chang P.V., Hao L., Offermanns S., Medzhitov R. (2014). The microbial metabolite butyrate regulates intestinal macrophage function via histone deacetylase inhibition. Proc. Natl. Acad. Sci. USA.

[35] Singh N., Thangaraju M., Prasad P.D., Martin P.M., Lambert N.A., Boettger T., Offermanns S., Ganapathy V. (2010). Blockade of Dendritic Cell Development by Bacterial Fermentation Products Butyrate and Propionate through a Transporter (Slc5a8)-dependent Inhibition of Histone Deacetylases. J. Biol. Chem..

[36] Smith P.M., Howitt M.R., Panikov N., Michaud M., Gallini C.A., Bohlooly Y.M., Glickman J.N., Garrett W.S. (2013). The microbial metabolites, short-chain fatty acids, regulate colonic Treg cell homeostasis. Science.

[37] Furusawa Y., Obata Y., Fukuda S., Endo T.A., Nakato G., Takahashi D., Nakanishi Y., Uetake C., Kato K., Kato T. (2013). Commensal microbe-derived butyrate induces the differentiation of colonic regulatory T cells. Nature.

[38] Atarashi K., Tanoue T., Oshima K., Suda W., Nagano Y., Nishikawa H., Fukuda S., Saito T., Narushima S., Hase K. (2013). Treg induction by a rationally selected mixture of Clostridia strains from the human microbiota. Nature.

[39] Arpaia N., Campbell C., Fan X.Y., Dikiy S., van der Veeken J., deRoos P., Liu H., Cross J.R., Pfeffer K., Coffer P.J. (2013). Metabolites produced by commensal bacteria promote peripheral regulatory T-cell generation. Nature.

[40] Park J., Kim M., Kang S.G., Jannasch A.H., Cooper B., Patterson J., Kim C.H. (2015). Short-chain fatty acids induce both effector and regulatory T cells by suppression of histone deacetylases and regulation of the mTOR-S6K pathway. Mucosal Immunol..

[41] He Y., Fu L., Li Y., Wang W., Gong M., Zhang J., Dong X., Huang J., Wang Q., Mackay C.R. (2021). Gut microbial metabolites facilitate anticancer therapy efficacy by modulating cytotoxic CD8(+) T cell immunity. Cell Metab..

[42] Spencer S.P., Fragiadakis G.K., Sonnenburg J.L. (2019). Pursuing Human-Relevant Gut Microbiota-Immune Interactions. Immunity.

[43] Singh N., Gurav A., Sivaprakasam S., Brady E., Padia R., Shi H., Thangaraju M., Prasad P.D., Manicassamy S., Munn D.H. (2014). Activation of Gpr109a, receptor for niacin and the commensal metabolite butyrate, suppresses colonic inflammation and carcinogenesis. Immunity.

[44] Sivaprakasam S., Gurav A., Paschall A.V., Coe G.L., Chaudhary K., Cai Y., Kolhe R., Martin P., Browning D., Huang L. (2016). An essential role of Ffar2 (Gpr43) in dietary fibre-mediated promotion of healthy composition of gut microbiota and suppression of intestinal carcinogenesis. Oncogenesis.

[45] Maslowski K.M., Vieira A.T., Ng A., Kranich J., Sierro F., Yu D., Schilter H.C., Rolph M.S., Mackay F., Artis D. (2009). Regulation of inflammatory responses by gut microbiota and chemoattractant receptor GPR43. Nature.

[46] Lavoie S., Chun E., Bae S., Brennan C.A., Gallini Comeau C.A., Lang J.K., Michaud M., Hoveyda H.R., Fraser G.L., Fuller M.H. (2020). Expression of Free Fatty Acid Receptor 2 by Dendritic Cells Prevents Their Expression of Interleukin 27 and Is Required for Maintenance of Mucosal Barrier and Immune Response Against Colorectal Tumors in Mice. Gastroenterology.

[47] Thangaraju M., Cresci G.A., Liu K., Ananth S., Gnanaprakasam J.P., Browning D.D., Mellinger J.D., Smith S.B., Digby G.J., Lambert N.A. (2009). GPR109A is a G-protein-coupled receptor for the bacterial fermentation product butyrate and functions as a tumor suppressor in colon. Cancer Res..

[48] Yang W., Yu T., Huang X., Bilotta A.J., Xu L., Lu Y., Sun J., Pan F., Zhou J., Zhang W. (2020). Intestinal microbiota-derived short-chain fatty acids regulation of immune cell IL-22 production and gut immunity. Nat. Commun..

[49] Di Sabatino A., Morera R., Ciccocioppo R., Cazzola P., Gotti S., Tinozzi F.P., Tinozzi S., Corazza G.R. (2005). Oral butyrate for mildly to moderately active Crohn’s disease. Aliment. Pharmacol. Ther..

[50] Cleophas M.C.P., Ratter J.M., Bekkering S., Quintin J., Schraa K., Stroes E.S., Netea M.G., Joosten L.A.B. (2019). Effects of oral butyrate supplementation on inflammatory potential of circulating peripheral blood mononuclear cells in healthy and obese males. Sci. Rep..

[51] Macia L., Tan J., Vieira A.T., Leach K., Stanley D., Luong S., Maruya M., Ian McKenzie C., Hijikata A., Wong C. (2015). Metabolite-sensing receptors GPR43 and GPR109A facilitate dietary fibre-induced gut homeostasis through regulation of the inflammasome. Nat. Commun..

[52] Fukuda S., Toh H., Hase K., Oshima K., Nakanishi Y., Yoshimura K., Tobe T., Clarke J.M., Topping D.L., Suzuki T. (2011). Bifidobacteria can protect from enteropathogenic infection through production of acetate. Nature.

[53] Waldschmitt N., Chamaillard M. (2015). Time for epithelial sensing of vitamin D to step into the limelight. Gut.

[54] Byndloss M.X., Olsan E.E., Rivera-Chavez F., Tiffany C.R., Cevallos S.A., Lokken K.L., Torres T.P., Byndloss A.J., Faber F., Gao Y. (2017). Microbiota-activated PPAR-gamma signaling inhibits dysbiotic Enterobacteriaceae expansion. Science.

[55] Tilg H., Adolph T.E., Gerner R.R., Moschen A.R. (2018). The Intestinal Microbiota in Colorectal Cancer. Cancer Cell.

[56] Yang K., Hou Y., Zhang Y., Liang H., Sharma A., Zheng W., Wang L., Torres R., Tatebe K., Chmura S.J. (2021). Suppression of local type I interferon by gut microbiota–derived butyrate impairs antitumor effects of ionizing radiation. J. Exp. Med..

[57] Behary J., Amorim N., Jiang X.T., Raposo A., Gong L., McGovern E., Ibrahim R., Chu F., Stephens C., Jebeili H. (2021). Gut microbiota impact on the peripheral immune response in non-alcoholic fatty liver disease related hepatocellular carcinoma. Nat. Commun..

[58] Belcheva A., Irrazabal T., Robertson S.J., Streutker C., Maughan H., Rubino S., Moriyama E.H., Copeland J.K., Kumar S., Green B. (2014). Gut Microbial Metabolism Drives Transformation of Msh2-Deficient Colon Epithelial Cells. Cell.

[59] Donohoe D.R., Collins L.B., Wali A., Bigler R., Sun W., Bultman S.J. (2012). The Warburg effect dictates the mechanism of butyrate-mediated histone acetylation and cell proliferation. Mol. Cell.

[60] Bultman S.J., Jobin C. (2014). Microbial-derived butyrate: An oncometabolite or tumor-suppressive metabolite?. Cell Host Microbe.

[61] Barker N., Ridgway R.A., van Es J.H., van de Wetering M., Begthel H., van den Born M., Danenberg E., Clarke A.R., Sansom O.J., Clevers H. (2009). Crypt stem cells as the cells-of-origin of intestinal cancer. Nature.

[62] Sphyris N., Hodder M.C., Sansom O.J. (2021). Subversion of Niche-Signalling Pathways in Colorectal Cancer: What Makes and Breaks the Intestinal Stem Cell. Cancers.

[63] Kaiko G.E., Ryu S.H., Koues O.I., Collins P.L., Solnica-Krezel L., Pearce E.J., Pearce E.L., Oltz E.M., Stappenbeck T.S. (2016). The Colonic Crypt Protects Stem Cells from Microbiota-Derived Metabolites. Cell.

[64] Cardona F., Andres-Lacueva C., Tulipani S., Tinahones F.J., Queipo-Ortuno M.I. (2013). Benefits of polyphenols on gut microbiota and implications in human health. J. Nutr. Biochem..

[65] Soldati L., Di Renzo L., Jirillo E., Ascierto P.A., Marincola F.M., De Lorenzo A. (2018). The influence of diet on anti-cancer immune responsiveness. J. Transl. Med..

[66] Li C., Ai G., Wang Y., Lu Q., Luo C., Tan L., Lin G., Liu Y., Li Y., Zeng H. (2020). Oxyberberine, a novel gut microbiota-mediated metabolite of berberine, possesses superior anti-colitis effect: Impact on intestinal epithelial barrier, gut microbiota profile and TLR4-MyD88-NF-kappaB pathway. Pharmacol. Res..

[67] Beyer-Sehlmeyer G., Glei M., Hartmann E., Hughes R., Persin C., Bohm V., Rowland I., Schubert R., Jahreis G., Pool-Zobel B.L. (2003). Butyrate is only one of several growth inhibitors produced during gut flora-mediated fermentation of dietary fibre sources. Br. J. Nutr..

[68] Duenas M., Munoz-Gonzalez I., Cueva C., Jimenez-Giron A., Sanchez-Patan F., Santos-Buelga C., Moreno-Arribas M.V., Bartolome B. (2015). A survey of modulation of gut microbiota by dietary polyphenols. BioMed Res. Int..

[69] Liu Y., Wang X., Chen Q., Luo L., Ma M., Xiao B., Zeng L. (2020). Camellia sinensis and Litsea coreana Ameliorate Intestinal Inflammation and Modulate Gut Microbiota in Dextran Sulfate Sodium-Induced Colitis Mice. Mol. Nutr. Food Res..

[70] Paivarinta E., Niku M., Maukonen J., Storvik M., Heiman-Lindh A., Saarela M., Pajari A.M., Mutanen M. (2016). Changes in intestinal immunity, gut microbiota, and expression of energy metabolism-related genes explain adenoma growth in bilberry and cloudberry-fed Apc(Min) mice. Nutr. Res..

[71] Saadatdoust Z., Pandurangan A.K., Ananda Sadagopan S.K., Mohd Esa N., Ismail A., Mustafa M.R. (2015). Dietary cocoa inhibits colitis associated cancer: A crucial involvement of the IL-6/STAT3 pathway. J. Nutr. Biochem..

[72] Bhattacharyya S., Md Sakib Hossain D., Mohanty S., Sankar Sen G., Chattopadhyay S., Banerjee S., Chakraborty J., Das K., Sarkar D., Das T. (2010). Curcumin reverses T cell-mediated adaptive immune dysfunctions in tumor-bearing hosts. Cell. Mol. Immunol..

[73] Kadosh E., Snir-Alkalay I., Venkatachalam A., May S., Lasry A., Elyada E., Zinger A., Shaham M., Vaalani G., Mernberger M. (2020). The gut microbiome switches mutant p53 from tumour-suppressive to oncogenic. Nature.

[74] Zhang Y., Weng Y., Gan H., Zhao X., Zhi F. (2018). *Streptococcus gallolyticus* conspires myeloid cells to promote tumorigenesis of inflammatory bowel disease. Biochem. Biophys. Res. Commun..

[75] Martel J., Ojcius D.M., Ko Y.F., Young J.D. (2020). Phytochemicals as Prebiotics and Biological Stress Inducers. Trends Biochem. Sci..

[76] Chen L., Jiang B., Zhong C., Guo J., Zhang L., Mu T., Zhang Q., Bi X. (2018). Chemoprevention of colorectal cancer by black raspberry anthocyanins involved the modulation of gut microbiota and SFRP2 demethylation. Carcinogenesis.

[77] Wu T.R., Lin C.S., Chang C.J., Lin T.L., Martel J., Ko Y.F., Ojcius D.M., Lu C.C., Young J.D., Lai H.C. (2019). Gut commensal *Parabacteroides goldsteinii* plays a predominant role in the anti-obesity effects of polysaccharides isolated from *Hirsutella sinensis*. Gut.

[78] Busbee P.B., Menzel L., Alrafas H.R., Dopkins N., Becker W., Miranda K., Tang C., Chatterjee S., Singh U., Nagarkatti M. (2020). Indole-3-carbinol prevents colitis and associated microbial dysbiosis in an IL-22-dependent manner. JCI Insight.

[79] Karthikkumar V., Sivagami G., Vinothkumar R., Rajkumar D., Nalini N. (2012). Modulatory efficacy of rosmarinic acid on premalignant lesions and antioxidant status in 1,2-dimethylhydrazine induced rat colon carcinogenesis. Environ. Toxicol. Pharmacol..

[80] Oliphant K., Allen-Vercoe E. (2019). Macronutrient metabolism by the human gut microbiome: Major fermentation by-products and their impact on host health. Microbiome.

[81] Roager H.M., Licht T.R. (2018). Microbial tryptophan catabolites in health and disease. Nat. Commun..

[82] Metidji A., Omenetti S., Crotta S., Li Y., Nye E., Ross E., Li V., Maradana M.R., Schiering C., Stockinger B. (2018). The Environmental Sensor AHR Protects from Inflammatory Damage by Maintaining Intestinal Stem Cell Homeostasis and Barrier Integrity. Immunity.

[83] Islam J., Sato S., Watanabe K., Watanabe T., Hirahara K., Aoyama Y., Tomita S., Aso H., Komai M., Shirakawa H. (2017). Dietary tryptophan alleviates dextran sodium sulfate-induced colitis through aryl hydrocarbon receptor in mice. J. Nutr. Biochem..

[84] Scott S.A., Fu J., Chang P.V. (2020). Microbial tryptophan metabolites regulate gut barrier function via the aryl hydrocarbon receptor. Proc. Natl. Acad. Sci. USA.

[85] Zelante T., Iannitti R.G., Cunha C., De Luca A., Giovannini G., Pieraccini G., Zecchi R., D’Angelo C., Massi-Benedetti C., Fallarino F. (2013). Tryptophan catabolites from microbiota engage aryl hydrocarbon receptor and balance mucosal reactivity via interleukin-22. Immunity.

[86] Kiss E.A., Vonarbourg C., Kopfmann S., Hobeika E., Finke D., Esser C., Diefenbach A. (2011). Natural aryl hydrocarbon receptor ligands control organogenesis of intestinal lymphoid follicles. Science.

[87] Lamas B., Richard M.L., Leducq V., Pham H.P., Michel M.L., Da Costa G., Bridonneau C., Jegou S., Hoffmann T.W., Natividad J.M. (2016). CARD9 impacts colitis by altering gut microbiota metabolism of tryptophan into aryl hydrocarbon receptor ligands. Nat. Med..

[88] Hou Q., Ye L., Liu H., Huang L., Yang Q., Turner J.R., Yu Q. (2018). Lactobacillus accelerates ISCs regeneration to protect the integrity of intestinal mucosa through activation of STAT3 signaling pathway induced by LPLs secretion of IL-22. Cell Death Differ..

[89] Gronke K., Hernandez P.P., Zimmermann J., Klose C.S.N., Kofoed-Branzk M., Guendel F., Witkowski M., Tizian C., Amann L., Schumacher F. (2019). Interleukin-22 protects intestinal stem cells against genotoxic stress. Nature.

[90] Venkatesh M., Mukherjee S., Wang H., Li H., Sun K., Benechet A.P., Qiu Z., Maher L., Redinbo M.R., Phillips R.S. (2014). Symbiotic bacterial metabolites regulate gastrointestinal barrier function via the xenobiotic sensor PXR and Toll-like receptor 4. Immunity.

[91] Chen J., Rao J.N., Zou T., Liu L., Marasa B.S., Xiao L., Zeng X., Turner D.J., Wang J.Y. (2007). Polyamines are required for expression of Toll-like receptor 2 modulating intestinal epithelial barrier integrity. Am. J. Physiol. Gastrointest. Liver Physiol..

[92] Liu L., Guo X., Rao J.N., Zou T., Xiao L., Yu T., Timmons J.A., Turner D.J., Wang J.Y. (2009). Polyamines regulate E-cadherin transcription through c-Myc modulating intestinal epithelial barrier function. Am. J. Physiol. Cell Physiol..

[93] Levy M., Thaiss C.A., Zeevi D., Dohnalova L., Zilberman-Schapira G., Mahdi J.A., David E., Savidor A., Korem T., Herzig Y. (2015). Microbiota-Modulated Metabolites Shape the Intestinal Microenvironment by Regulating NLRP6 Inflammasome Signaling. Cell.

[94] Hardbower D.M., Asim M., Luis P.B., Singh K., Barry D.P., Yang C., Steeves M.A., Cleveland J.L., Schneider C., Piazuelo M.B. (2017). Ornithine decarboxylase regulates M1 macrophage activation and mucosal inflammation via histone modifications. Proc. Natl. Acad. Sci. USA.

[95] Singh K., Coburn L.A., Asim M., Barry D.P., Allaman M.M., Shi C., Washington M.K., Luis P.B., Schneider C., Delgado A.G. (2018). Ornithine Decarboxylase in Macrophages Exacerbates Colitis and Promotes Colitis-Associated Colon Carcinogenesis by Impairing M1 Immune Responses. Cancer Res..

[96] Hasko G., Kuhel D.G., Marton A., Nemeth Z.H., Deitch E.A., Szabo C. (2000). Spermine differentially regulates the production of interleukin-12 p40 and interleukin-10 and suppresses the release of the T helper 1 cytokine interferon-gamma. Shock.

[97] Miller-Fleming L., Olin-Sandoval V., Campbell K., Ralser M. (2015). Remaining Mysteries of Molecular Biology: The Role of Polyamines in the Cell. J. Mol. Biol..

[98] Hayes C.S., Shicora A.C., Keough M.P., Snook A.E., Burns M.R., Gilmour S.K. (2014). Polyamine-blocking therapy reverses immunosuppression in the tumor microenvironment. Cancer Immunol. Res..

[99] Alexander E.T., Mariner K., Donnelly J., Phanstiel O.t., Gilmour S.K. (2020). Polyamine Blocking Therapy Decreases Survival of Tumor-Infiltrating Immunosuppressive Myeloid Cells and Enhances the Antitumor Efficacy of PD-1 Blockade. Mol. Cancer Ther..

[100] Mondanelli G., Bianchi R., Pallotta M.T., Orabona C., Albini E., Iacono A., Belladonna M.L., Vacca C., Fallarino F., Macchiarulo A. (2017). A Relay Pathway between Arginine and Tryptophan Metabolism Confers Immunosuppressive Properties on Dendritic Cells. Immunity.

[101] Puleston D.J., Buck M.D., Klein Geltink R.I., Kyle R.L., Caputa G., O’Sullivan D., Cameron A.M., Castoldi A., Musa Y., Kabat A.M. (2019). Polyamines and eIF5A Hypusination Modulate Mitochondrial Respiration and Macrophage Activation. Cell Metab..

[102] Pietrocola F., Pol J., Vacchelli E., Rao S., Enot D.P., Baracco E.E., Levesque S., Castoldi F., Jacquelot N., Yamazaki T. (2016). Caloric Restriction Mimetics Enhance Anticancer Immunosurveillance. Cancer Cell.

[103] Johnson C.H., Dejea C.M., Edler D., Hoang L.T., Santidrian A.F., Felding B.H., Ivanisevic J., Cho K., Wick E.C., Hechenbleikner E.M. (2015). Metabolism links bacterial biofilms and colon carcinogenesis. Cell Metab..

[104] Goodwin A.C., Destefano Shields C.E., Wu S., Huso D.L., Wu X., Murray-Stewart T.R., Hacker-Prietz A., Rabizadeh S., Woster P.M., Sears C.L. (2011). Polyamine catabolism contributes to enterotoxigenic Bacteroides fragilis-induced colon tumorigenesis. Proc. Natl. Acad. Sci. USA.

[105] Attene-Ramos M.S., Nava G.M., Muellner M.G., Wagner E.D., Plewa M.J., Gaskins H.R. (2010). DNA damage and toxicogenomic analyses of hydrogen sulfide in human intestinal epithelial FHs 74 Int cells. Environ. Mol. Mutagen..

[106] Devkota S., Wang Y., Musch M.W., Leone V., Fehlner-Peach H., Nadimpalli A., Antonopoulos D.A., Jabri B., Chang E.B. (2012). Dietary-fat-induced taurocholic acid promotes pathobiont expansion and colitis in Il10^−/−^ mice. Nature.

[107] Ijssennagger N., Belzer C., Hooiveld G.J., Dekker J., van Mil S.W., Muller M., Kleerebezem M., van der Meer R. (2015). Gut microbiota facilitates dietary heme-induced epithelial hyperproliferation by opening the mucus barrier in colon. Proc. Natl. Acad. Sci. USA.

[108] Ridlon J.M., Wolf P.G., Gaskins H.R. (2016). Taurocholic acid metabolism by gut microbes and colon cancer. Gut Microbes.

[109] Beeckmans D., Riethorst D., Augustijns P., Vanuytsel T., Farre R., Tack J., Vanheel H. (2018). Altered duodenal bile salt concentration and receptor expression in functional dyspepsia. United Eur. Gastroenterol. J..

[110] Spigelman A.D., Owen R.W., Hill M.J., Phillips R.K. (1991). Biliary bile acid profiles in familial adenomatous polyposis. Br. J. Surg..

[111] Liu H., Tian R., Wang H., Feng S., Li H., Xiao Y., Luan X., Zhang Z., Shi N., Niu H. (2020). Gut microbiota from coronary artery disease patients contributes to vascular dysfunction in mice by regulating bile acid metabolism and immune activation. J. Transl. Med..

[112] Bhargava P., Smith M.D., Mische L., Harrington E., Fitzgerald K.C., Martin K., Kim S., Reyes A.A., Gonzalez-Cardona J., Volsko C. (2020). Bile acid metabolism is altered in multiple sclerosis and supplementation ameliorates neuroinflammation. J. Clin. Investig..

[113] Imray C.H., Radley S., Davis A., Barker G., Hendrickse C.W., Donovan I.A., Lawson A.M., Baker P.R., Neoptolemos J.P. (1992). Faecal unconjugated bile acids in patients with colorectal cancer or polyps. Gut.

[114] Fiorucci S., Biagioli M., Zampella A., Distrutti E. (2018). Bile Acids Activated Receptors Regulate Innate Immunity. Front. Immunol..

[115] Pascual G., Fong A.L., Ogawa S., Gamliel A., Li A.C., Perissi V., Rose D.W., Willson T.M., Rosenfeld M.G., Glass C.K. (2005). A SUMOylation-dependent pathway mediates transrepression of inflammatory response genes by PPAR-gamma. Nature.

[116] Vavassori P., Mencarelli A., Renga B., Distrutti E., Fiorucci S. (2009). The bile acid receptor FXR is a modulator of intestinal innate immunity. J. Immunol..

[117] Guo C., Qi H., Yu Y., Zhang Q., Su J., Yu D., Huang W., Chen W.D., Wang Y.D. (2015). The G-Protein-Coupled Bile Acid Receptor Gpbar1 (TGR5) Inhibits Gastric Inflammation Through Antagonizing NF-kappaB Signaling Pathway. Front. Pharmacol..

[118] Gadaleta R.M., van Erpecum K.J., Oldenburg B., Willemsen E.C., Renooij W., Murzilli S., Klomp L.W., Siersema P.D., Schipper M.E., Danese S. (2011). Farnesoid X receptor activation inhibits inflammation and preserves the intestinal barrier in inflammatory bowel disease. Gut.

[119] Cipriani S., Mencarelli A., Chini M.G., Distrutti E., Renga B., Bifulco G., Baldelli F., Donini A., Fiorucci S. (2011). The bile acid receptor GPBAR-1 (TGR5) modulates integrity of intestinal barrier and immune response to experimental colitis. PLoS ONE.

[120] Sinha S.R., Haileselassie Y., Nguyen L.P., Tropini C., Wang M., Becker L.S., Sim D., Jarr K., Spear E.T., Singh G. (2020). Dysbiosis-Induced Secondary Bile Acid Deficiency Promotes Intestinal Inflammation. Cell Host Microbe.

[121] Campbell C., McKenney P.T., Konstantinovsky D., Isaeva O.I., Schizas M., Verter J., Mai C., Jin W.B., Guo C.J., Violante S. (2020). Bacterial metabolism of bile acids promotes generation of peripheral regulatory T cells. Nature.

[122] Song X., Sun X., Oh S.F., Wu M., Zhang Y., Zheng W., Geva-Zatorsky N., Jupp R., Mathis D., Benoist C. (2020). Microbial bile acid metabolites modulate gut RORgamma(+) regulatory T cell homeostasis. Nature.

[123] Franzosa E.A., Sirota-Madi A., Avila-Pacheco J., Fornelos N., Haiser H.J., Reinker S., Vatanen T., Hall A.B., Mallick H., McIver L.J. (2019). Gut microbiome structure and metabolic activity in inflammatory bowel disease. Nat. Microbiol..

[124] Duboc H., Rajca S., Rainteau D., Benarous D., Maubert M.A., Quervain E., Thomas G., Barbu V., Humbert L., Despras G. (2013). Connecting dysbiosis, bile-acid dysmetabolism and gut inflammation in inflammatory bowel diseases. Gut.

[125] Wirbel J., Pyl P.T., Kartal E., Zych K., Kashani A., Milanese A., Fleck J.S., Voigt A.Y., Palleja A., Ponnudurai R. (2019). Meta-analysis of fecal metagenomes reveals global microbial signatures that are specific for colorectal cancer. Nat. Med..

[126] Ou J., Carbonero F., Zoetendal E.G., DeLany J.P., Wang M., Newton K., Gaskins H.R., O’Keefe S.J. (2013). Diet, microbiota, and microbial metabolites in colon cancer risk in rural Africans and African Americans. Am. J. Clin. Nutr..

[127] Farhana L., Nangia-Makker P., Arbit E., Shango K., Sarkar S., Mahmud H., Hadden T., Yu Y., Majumdar A.P. (2016). Bile acid: A potential inducer of colon cancer stem cells. Stem Cell Res. Ther..

[128] Marzano M., Fosso B., Piancone E., Defazio G., Pesole G., De Robertis M. (2021). Stem Cell Impairment at the Host-Microbiota Interface in Colorectal Cancer. Cancers.

[129] Fu T., Coulter S., Yoshihara E., Oh T.G., Fang S., Cayabyab F., Zhu Q., Zhang T., Leblanc M., Liu S. (2019). FXR Regulates Intestinal Cancer Stem Cell Proliferation. Cell.

[130] Sorrentino G., Perino A., Yildiz E., El Alam G., Bou Sleiman M., Gioiello A., Pellicciari R., Schoonjans K. (2020). Bile Acids Signal via TGR5 to Activate Intestinal Stem Cells and Epithelial Regeneration. Gastroenterology.

[131] Bernstein C., Holubec H., Bhattacharyya A.K., Nguyen H., Payne C.M., Zaitlin B., Bernstein H. (2011). Carcinogenicity of deoxycholate, a secondary bile acid. Arch. Toxicol..

[132] Hegyi P., Maleth J., Walters J.R., Hofmann A.F., Keely S.J. (2018). Guts and Gall: Bile Acids in Regulation of Intestinal Epithelial Function in Health and Disease. Physiol. Rev..

[133] Murakami Y., Tanabe S., Suzuki T. (2016). High-fat Diet-induced Intestinal Hyperpermeability is Associated with Increased Bile Acids in the Large Intestine of Mice. J. Food Sci..

[134] Barrasa J.I., Olmo N., Lizarbe M.A., Turnay J. (2013). Bile acids in the colon, from healthy to cytotoxic molecules. Toxicol. In Vitro.

[135] Zhao S., Gong Z., Zhou J., Tian C., Gao Y., Xu C., Chen Y., Cai W., Wu J. (2016). Deoxycholic Acid Triggers NLRP3 Inflammasome Activation and Aggravates DSS-Induced Colitis in Mice. Front. Immunol..

[136] Modica S., Murzilli S., Salvatore L., Schmidt D.R., Moschetta A. (2008). Nuclear Bile Acid Receptor FXR Protects against Intestinal Tumorigenesis. Cancer Res..

[137] Castellarin M., Warren R.L., Freeman J.D., Dreolini L., Krzywinski M., Strauss J., Barnes R., Watson P., Allen-Vercoe E., Moore R.A. (2012). *Fusobacterium nucleatum* infection is prevalent in human colorectal carcinoma. Genome Res..

[138] Kostic A.D., Gevers D., Pedamallu C.S., Michaud M., Duke F., Earl A.M., Ojesina A.I., Jung J., Bass A.J., Tabernero J. (2012). Genomic analysis identifies association of Fusobacterium with colorectal carcinoma. Genome Res..

[139] Bullman S., Pedamallu C.S., Sicinska E., Claney T.E., Zhang X.Y., Cai D.N., Neuberg D., Huang K., Guevara F., Nelson T. (2017). Analysis of Fusobacterium persistence and antibiotic response in colorectal cancer. Science.

[140] Rubinstein M.R., Wang X.W., Liu W.D., Hao Y.J., Cai G.F., Han Y.P.W. (2013). *Fusobacterium nucleatum* Promotes Colorectal Carcinogenesis by Modulating E-Cadherin/beta-Catenin Signaling via its FadA Adhesin. Cell Host Microbe.

[141] Kostic A.D., Chun E.Y., Robertson L., Glickman J.N., Gallini C.A., Michaud M., Clancy T.E., Chung D.C., Lochhead P., Hold G.L. (2013). *Fusobacterium nucleatum* Potentiates Intestinal Tumorigenesis and Modulates the Tumor-Immune Microenvironment. Cell Host Microbe.

[142] Gur C., Ibrahim Y., Isaacson B., Yamin R., Abed J., Gamliel M., Enk J., Bar-On Y., Stanietsky-Kaynan N., Coppenhagen-Glazer S. (2015). Binding of the Fap2 protein of *Fusobacterium nucleatum* to human inhibitory receptor TIGIT protects tumors from immune cell attack. Immunity.

[143] Mima K., Sukawa Y., Nishihara R., Qian Z.R., Yamauchi M., Inamura K., Kim S.A., Masuda A., Nowak J.A., Nosho K. (2015). *Fusobacterium nucleatum* and T Cells in Colorectal Carcinoma. JAMA Oncol..

[144] Nosho K., Sukawa Y., Adachi Y., Ito M., Mitsuhashi K., Kurihara H., Kanno S., Yamamoto I., Ishigami K., Igarashi H. (2016). Association of *Fusobacterium nucleatum* with immunity and molecular alterations in colorectal cancer. World J. Gastroenterol..

[145] Yu T.C., Guo F.F., Yu Y.N., Sun T.T., Ma D., Han J.X., Qian Y., Kryczek I., Sun D.F., Nagarsheth N. (2017). *Fusobacterium nucleatum* Promotes Chemoresistance to Colorectal Cancer by Modulating Autophagy. Cell.

[146] Yang Y.Z., Weng W.H., Peng J.J., Hong L.M., Yang L., Toiyama Y., Gao R.Y., Liu M.F., Yin M.M., Pan C. (2017). *Fusobacterium nucleatum* Increases Proliferation of Colorectal Cancer Cells and Tumor Development in Mice by Activating Toll-Like Receptor 4 Signaling to Nuclear Factor-kappa B, and Up-regulating Expression of MicroRNA-21. Gastroenterology.

[147] Dai Z., Coker O.O., Nakatsu G., Wu W.K.K., Zhao L., Chen Z., Chan F.K.L., Kristiansen K., Sung J.J.Y., Wong S.H. (2018). Multi-cohort analysis of colorectal cancer metagenome identified altered bacteria across populations and universal bacterial markers. Microbiome.

[148] Boleij A., Hechenbleikner E.M., Goodwin A.C., Badani R., Stein E.M., Lazarev M.G., Ellis B., Carroll K.C., Albesiano E., Wick E.C. (2015). The Bacteroides fragilis Toxin Gene Is Prevalent in the Colon Mucosa of Colorectal Cancer Patients. Clin. Infect. Dis..

[149] Wu S., Rhee K.J., Albesiano E., Rabizadeh S., Wu X., Yen H.R., Huso D.L., Brancati F.L., Wick E., McAllister F. (2009). A human colonic commensal promotes colon tumorigenesis via activation of T helper type 17 T cell responses. Nat. Med..

[150] Thiele Orberg E., Fan H., Tam A.J., Dejea C.M., Destefano Shields C.E., Wu S., Chung L., Finard B.B., Wu X., Fathi P. (2017). The myeloid immune signature of enterotoxigenic *Bacteroides fragilis*-induced murine colon tumorigenesis. Mucosal Immunol..

[151] Chung L., Thiele Orberg E., Geis A.L., Chan J.L., Fu K., DeStefano Shields C.E., Dejea C.M., Fathi P., Chen J., Finard B.B. (2018). *Bacteroides fragilis* Toxin Coordinates a Pro-carcinogenic Inflammatory Cascade via Targeting of Colonic Epithelial Cells. Cell Host Microbe.

[152] Nguyen H.T., Dalmasso G., Muller S., Carriere J., Seibold F., Darfeuille-Michaud A. (2014). Crohn’s disease-associated adherent invasive *Escherichia coli* modulate levels of microRNAs in intestinal epithelial cells to reduce autophagy. Gastroenterology.

[153] Dejea C.M., Fathi P., Craig J.M., Boleij A., Taddese R., Geis A.L., Wu X., DeStefano Shields C.E., Hechenbleikner E.M., Huso D.L. (2018). Patients with familial adenomatous polyposis harbor colonic biofilms containing tumorigenic bacteria. Science.

[154] Pleguezuelos-Manzano C., Puschhof J., Rosendahl Huber A., van Hoeck A., Wood H.M., Nomburg J., Gurjao C., Manders F., Dalmasso G., Stege P.B. (2020). Mutational signature in colorectal cancer caused by genotoxic pks(+) *E. coli*. Nature.

[155] Tsoi H., Chu E.S.H., Zhang X., Sheng J., Nakatsu G., Ng S.C., Chan A.W.H., Chan F.K.L., Sung J.J.Y., Yu J. (2017). *Peptostreptococcus anaerobius* Induces Intracellular Cholesterol Biosynthesis in Colon Cells to Induce Proliferation and Causes Dysplasia in Mice. Gastroenterology.

[156] Long X., Wong C.C., Tong L., Chu E.S.H., Ho Szeto C., Go M.Y.Y., Coker O.O., Chan A.W.H., Chan F.K.L., Sung J.J.Y. (2019). *Peptostreptococcus anaerobius* promotes colorectal carcinogenesis and modulates tumour immunity. Nat. Microbiol..

[157] Mager L.F., Burkhard R., Pett N., Cooke N.C.A., Brown K., Ramay H., Paik S., Stagg J., Groves R.A., Gallo M. (2020). Microbiome-derived inosine modulates response to checkpoint inhibitor immunotherapy. Science.

[158] Shalapour S., Karin M. (2020). Cruel to Be Kind: Epithelial, Microbial, and Immune Cell Interactions in Gastrointestinal Cancers. Annu. Rev. Immunol..

[159] Maisonneuve C., Tsang D.K.L., Foerster E.G., Robert L.M., Mukherjee T., Prescott D., Tattoli I., Lemire P., Winer D.A., Winer S. (2021). Nod1 promotes colorectal carcinogenesis by regulating the immunosuppressive functions of tumor-infiltrating myeloid cells. Cell Rep..

[160] Li Q., Hu W., Liu W.X., Zhao L.Y., Huang D., Liu X.D., Chan H., Zhang Y., Zeng J.D., Coker O.O. (2020). *Streptococcus thermophilus* inhibits colorectal tumorigenesis through secreting beta-galactosidase. Gastroenterology.

[161] Bingham S.A., Day N.E., Luben R., Ferrari P., Slimani N., Norat T., Clavel-Chapelon F., Kesse E., Nieters A., Boeing H. (2003). Dietary fibre in food and protection against colorectal cancer in the European Prospective Investigation into Cancer and Nutrition (EPIC): An observational study. Lancet.

[162] Murphy N., Norat T., Ferrari P., Jenab M., Bueno-de-Mesquita B., Skeie G., Dahm C.C., Overvad K., Olsen A., Tjonneland A. (2012). Dietary fibre intake and risks of cancers of the colon and rectum in the European prospective investigation into cancer and nutrition (EPIC). PLoS ONE.

[163] Song M., Wu K., Meyerhardt J.A., Ogino S., Wang M., Fuchs C.S., Giovannucci E.L., Chan A.T. (2018). Fiber Intake and Survival After Colorectal Cancer Diagnosis. JAMA Oncol..

[164] Schmit S.L., Rennert H.S., Rennert G., Gruber S.B. (2016). Coffee Consumption and the Risk of Colorectal Cancer. Cancer Epidemiol. Biomark. Prev..

[165] Hu Y., Ding M., Yuan C., Wu K., Smith-Warner S.A., Hu F.B., Chan A.T., Meyerhardt J.A., Ogino S., Fuchs C.S. (2018). Association between Coffee Intake after Diagnosis of Colorectal Cancer and Reduced Mortality. Gastroenterology.

[166] Mackintosh C., Yuan C., Ou F.S., Zhang S., Niedzwiecki D., Chang I.W., O’Neil B.H., Mullen B.C., Lenz H.J., Blanke C.D. (2020). Association of Coffee Intake with Survival in Patients with Advanced or Metastatic Colorectal Cancer. JAMA Oncol..

